# Post-traumatic Ossifying Labyrinthitis: A Case Report and Review of the Literature

**DOI:** 10.7759/cureus.93289

**Published:** 2025-09-26

**Authors:** Firas Oueslati, Sonia Esseghaier, Chiraz Halwani, Moncef Aloui, Younes Arous

**Affiliations:** 1 Department of Diagnostic Radiology, Hôpital Militaire Principal d'Instruction de Tunis, Tunis, TUN; 2 Department of Otorhinolaryngology, Hôpital Militaire Principal d'Instruction de Tunis, Tunis, TUN; 3 Department of Diagnostic Radiology, Hôpital Militaire Principal d'Instruction de Tunis, TUNIS, TUN

**Keywords:** cochlear implant, inner ear injury, labyrinthitis ossificans, temporal bone trauma, vertigo

## Abstract

Labyrinthitis ossificans is a rare but serious complication that can occur following inner ear trauma, often resulting in irreversible sensorineural hearing loss. We present the case of a 22-year-old male who developed progressive sensorineural hearing loss after a motor vehicle accident. High-resolution CT and MRI revealed ossification within the membranous labyrinth, consistent with labyrinthitis ossificans. This case highlights the importance of early radiologic assessment in post-traumatic hearing loss, as timely diagnosis is essential for optimizing cochlear implantation outcomes and preserving auditory function in patients with severe inner ear damage.

## Introduction

Labyrinthitis ossificans is a pathological condition characterized by the replacement of the delicate membranous labyrinth with fibrous tissue and bone, typically as a consequence of severe inner ear insult. It most commonly develops following bacterial meningitis but can also occur secondary to viral infections, autoimmune inner ear disease, or temporal bone trauma [1]. The resulting ossification can significantly compromise cochlear implant candidacy due to obstruction of the cochlear lumen, making early diagnosis crucial. High-resolution imaging techniques such as CT and MRI play a vital role in identifying the extent and stage of ossification, thereby guiding appropriate clinical and surgical decision-making [2]. This report presents a rare post-traumatic case of labyrinthitis ossificans, highlighting the importance of timely radiologic assessment in patients with progressive hearing loss after head injury.

## Case presentation

**Figure 1:**
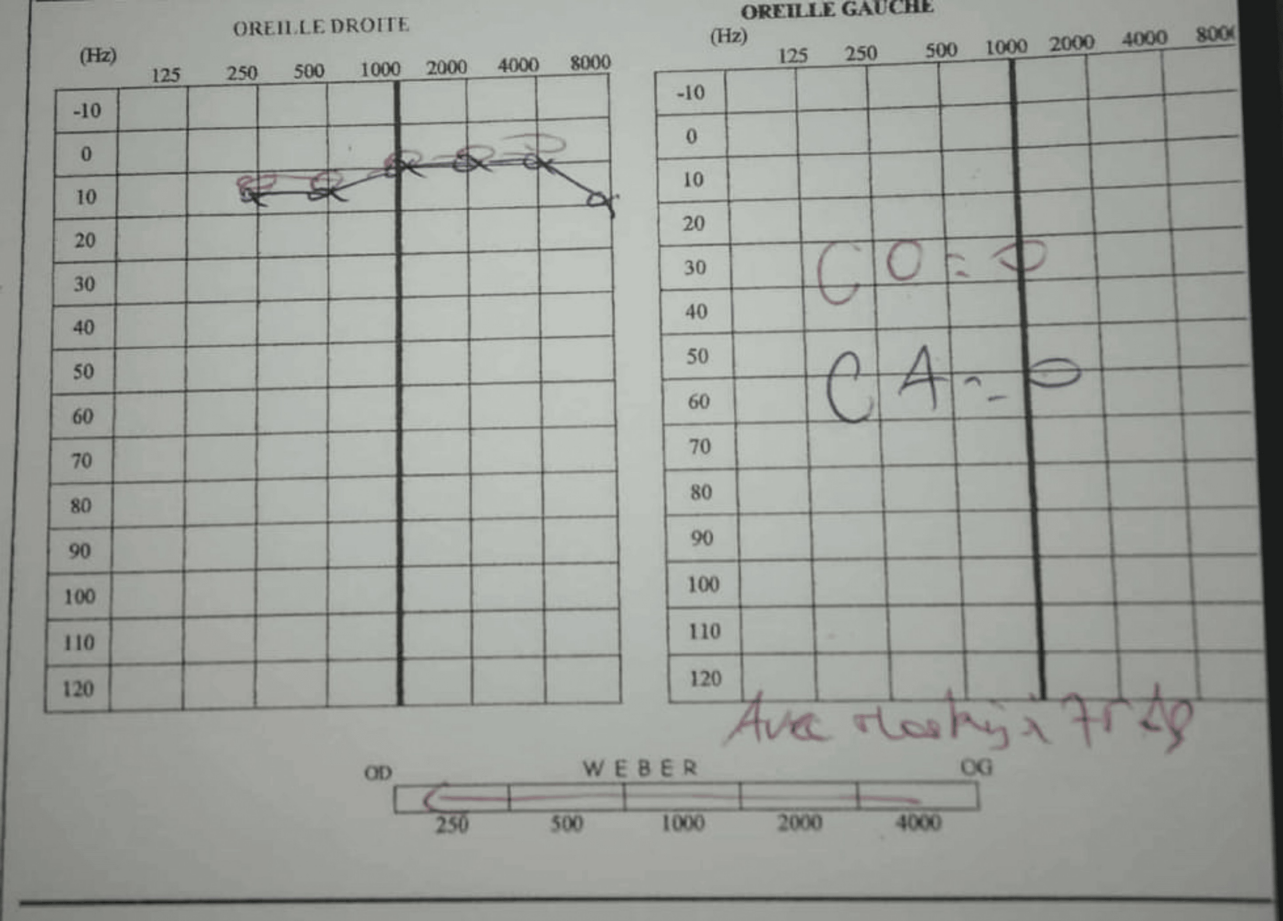
Audiogram showing left anacusis

A 22-year-old male, without any medical history, who had a car accident resulting in a traumatic brain injury, was initially admitted to the ICU after performing a cerebral computed tomography, revealing a left fronto-temporal extra-axial hematoma and multiple cranial fractures, particularly a left temporal bone fracture with complete otomastoid opacification. He underwent an evacuation of an extra-axial hematoma at the neurosurgery department, and the patient was discharged after being hospitalized for more than one month. He was complaining afterwards about vertigo and progressive hearing loss without any improvement with symptomatic treatment. An audiogram showed total sensorineural hearing loss (Figure 1).

**Figure 2:**
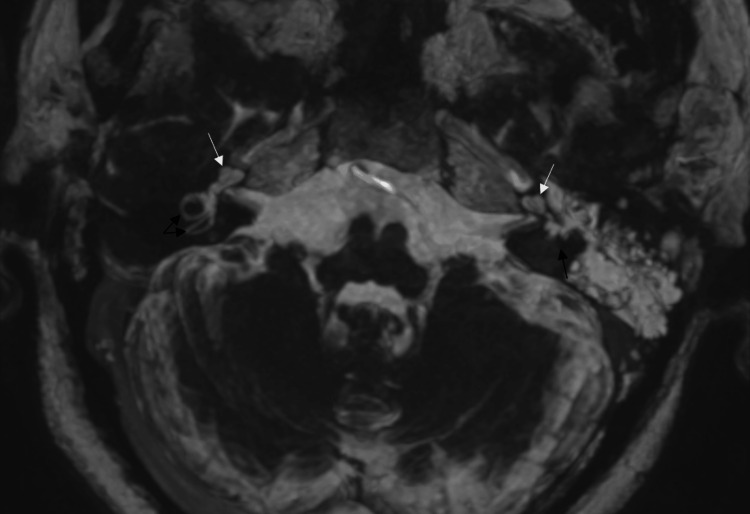
Axial T2-weighted MR images following MIP reconstruction; signal loss from all left three semi-circular canals Black arrows: semi-circular canals; white arrows: cochlea MIP: maximum intensity projection

High-resolution three-dimensional T2-weighted MR imaging using a fast spin echo sequence was performed six months post-trauma, revealing complete signal loss of fluid within the membranous labyrinth in all three semicircular canals (Figure 2).

**Figure 3:**
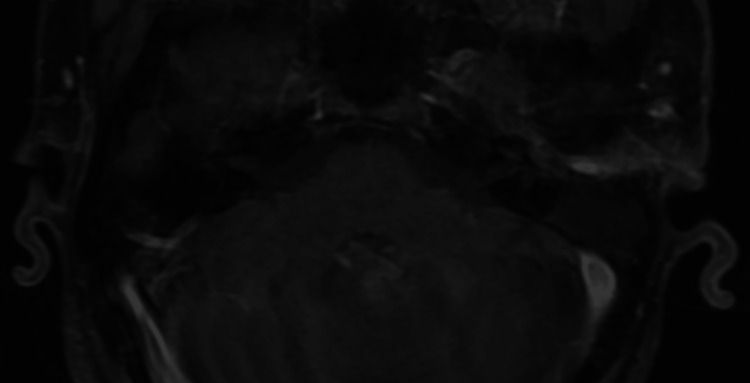
Post-contrast T1-weighted sequence; no enhancement observed in the anterior or posterior portions of the labyrinth

Post-contrast MRI sequence did not show any enhancement in the labyrinth (Figure 3).

**Figure 4:**
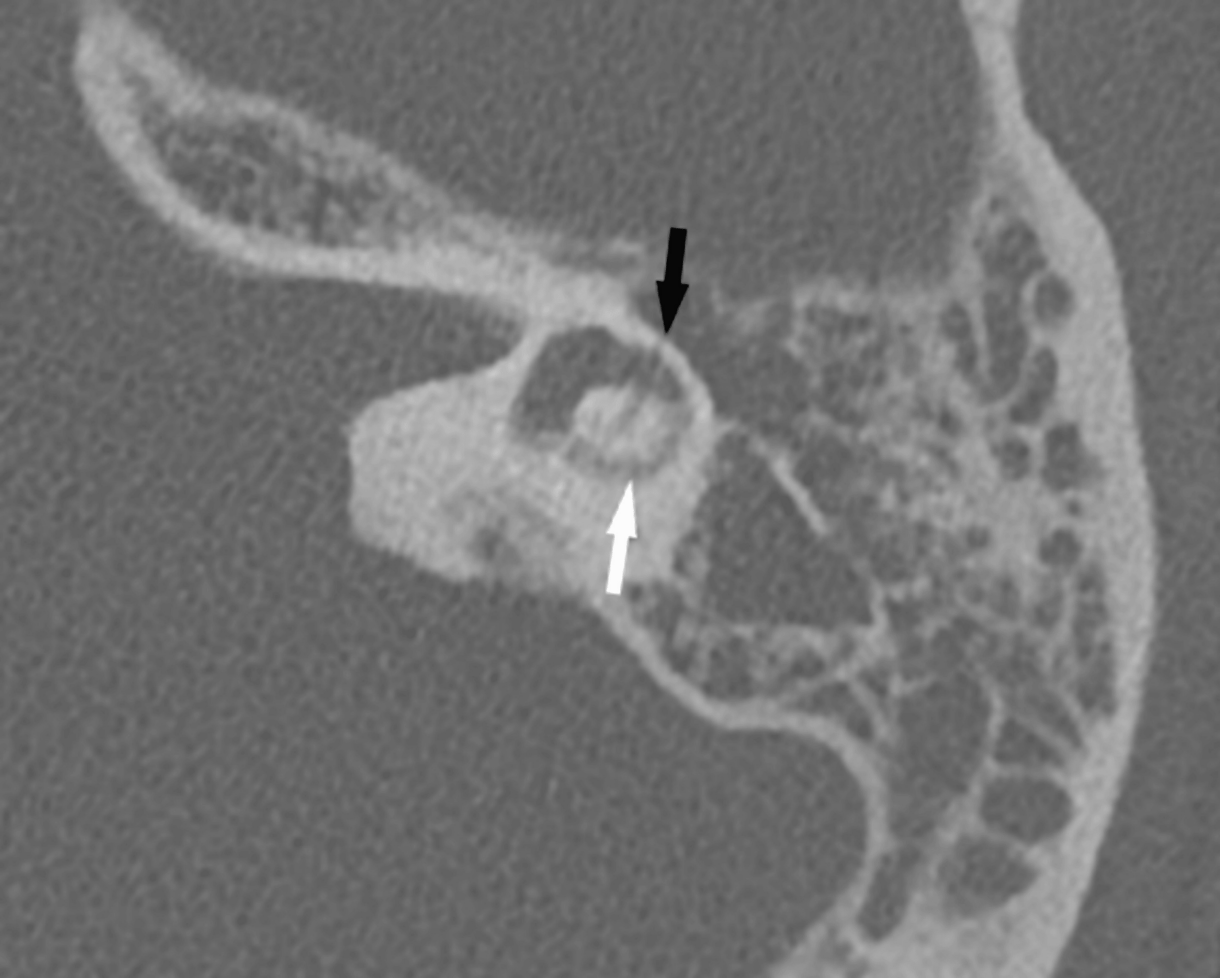
High-resolution axial CT scan of the left temporal bone; fracture involving the lateral semi-circular canal associated with ossification Black arrow: fracture; white arrow: mild ossification of the lateral canal

A complementary petrous temporal bone high-resolution computed tomography showed hyperattenuation in the lateral semicircular canal due to mild ossification (Figure 4).

The superior and posterior semicircular canals demonstrated normal density. The transverse temporal fracture with a translabyrinthine course involving the lateral semicircular canal remained visible. No associated ossicular dislocation was identified. The youth did not show any clinical improvement despite corticosteroid therapy and was subsequently evaluated for a cochlear implant as a last therapeutic option.

## Discussion

This case illustrates a rare instance of post-traumatic labyrinthitis ossificans, identified several months after a severe head injury involving a translabyrinthine temporal bone fracture. The patient initially presented with life-threatening intracranial injuries, which delayed the evaluation of vestibulocochlear function. The progressive onset of vertigo and sensorineural hearing loss, unresponsive to medical treatment, prompted delayed imaging, ultimately revealing ossification within the lateral semicircular canal. These findings underscore the importance of maintaining a high index of suspicion for inner ear damage in patients with temporal bone fractures, even when initial imaging and clinical management are focused on neurosurgical priorities. The physiopathology underlying post-traumatic labyrinthitis ossificans involves initial damage to the delicate membranous labyrinth caused by mechanical forces, such as temporal bone fractures or blunt head trauma. This damage induces an inflammatory response leading to fibrosis and subsequent ossification within the cochlear and vestibular compartments. The basal turn of the cochlea is the site most commonly affected by fibrosis and ossification in patients with prior temporal bone surgery; however, the precise location may differ depending on the route of inflammatory entry or dissemination, and all etiologies combined, semi-circular canals were most severely affected [3,4]. The ossification process typically progresses over weeks to months, as in our patient, resulting in irreversible sensorineural hearing loss and vestibular dysfunction. Early diagnosis is crucial but challenging. Imaging modalities play a pivotal role: high-resolution computed tomography allows visualization of bony labyrinth ossification. The progression of labyrinthitis ossificans is typically divided into three stages: an initial acute phase, followed by fibrotic changes, and ultimately ossification [5]. Axial 3D T2-weighted sequences were predominantly used to assess regions of abnormally low T2 signal within the membranous labyrinth [2]. In our case, signal extinction in all left canals contrasts with ossification only of the lateral semi-circular canal, confirming an early stage of ossification. In fact, MRI in the acute phase shows a hyperintense signal on T2-weighted images in the labyrinth, indicating inflammatory edema with marked enhancement after gadolinium contrast injection. In the fibrosis phase, a gradual decrease of T2 hyperintensity appears as edema subsides and a reduction of enhancement on T1 post-contrast. T2 hyperintensity is finally replaced by marked hypointensity due to ossification, and no more enhancement after contrast injection. Still, ossification is more clearly seen on a CT scan, but on MRI, there is signal loss related to mineralization [3].

In the setting of total sensorineural hearing loss, and in the absence of radiological involvement of the cochlea on either CT or MRI, as in our patient, the most likely etiologies include a posterior labyrinth fracture with concussion of the anterior labyrinth, a resorbed intracochlear hemorrhage, or a rupture of Reissner's membrane.

Treatment options remain limited. Hearing rehabilitation through hearing aids may be insufficient in cases of extensive ossification, and cochlear implantation has emerged as an effective intervention for restoring hearing in such patients. Incomplete insertion of the cochlear implant electrode may, in some instances, be attributed to intraluminal obstructions, such as osseous changes associated with labyrinthitis ossificans, otosclerosis, or temporal bone fractures, as well as soft tissue presence within the scala [6]. Therefore, early recognition of labyrinthitis ossificans in the post-traumatic setting is critical to optimize timing for cochlear implantation before extensive ossification occurs. In comparison with previous reports, our case underscores the importance of considering labyrinthitis ossificans in patients presenting with delayed-onset hearing loss following temporal bone trauma. A multidisciplinary approach involving otolaryngologists and radiologists is essential for accurate diagnosis and management.

## Conclusions

Post-traumatic ossifying labyrinthitis is a rare but significant complication that can lead to irreversible sensorineural hearing loss and vestibular dysfunction. This case underscores the critical role of early and appropiate imaging modalities. Prompt identification allows for better prognosis and timely cochlear implantation before complete ossification limits the procedure.
